# Associations between Periodontitis, COVID-19, and Cardiometabolic Complications: Molecular Mechanisms and Clinical Evidence

**DOI:** 10.3390/metabo13010040

**Published:** 2022-12-26

**Authors:** Giuseppe Mainas, Luigi Nibali, Mark Ide, Wael Al Mahmeed, Khalid Al-Rasadi, Kamila Al-Alawi, Maciej Banach, Yajnavalka Banerjee, Antonio Ceriello, Mustafa Cesur, Francesco Cosentino, Alberto Firenze, Massimo Galia, Su-Yen Goh, Andrej Janež, Sanjay Kalra, Nitin Kapoor, Peter Kempler, Nader Lessan, Paulo Lotufo, Nikolaos Papanas, Ali A. Rizvi, Amirhossein Sahebkar, Raul D. Santos, Anca P. Stoian, Peter P. Toth, Vijay Viswanathan, Manfredi Rizzo

**Affiliations:** 1 Periodontology Unit, Centre for Host-Microbiome Interactions, Dental Institute, King’s College London, London SE1 9RT, UK; 2 Heart and Vascular Institute, Cleveland Clinic, Abu Dhabi P.O. Box 112412, United Arab Emirates; 3 Medical Research Center, Sultan Qaboos University, Muscat 113, Oman; 4 Department of Training and Studies, Royal Hospital, Ministry of Health, Muscat 113, Oman; 5 Department of Preventive Cardiology and Lipidology, Medical University of Lodz (MUL), 90419 Lodz, Poland; 6 Polish Mother’s Memorial Hospital Research Institute (PMMHRI), 93338 Lodz, Poland; 7 Cardiovascular Research Centre, University of Zielona Gora, 65417 Zielona Gora, Poland; 8 Department of Biochemistry, Mohamed Bin Rashid University, Dubai 505055, United Arab Emirates; 9 IRCCS MultiMedica, 20099 Milan, Italy; 10 Clinic of Endocrinology, Ankara Güven Hospital, 06540 Ankara, Turkey; 11 Unit of Cardiology, Karolinska Institute and Karolinska University Hospital, University of Stockholm, 17177 Stockholm, Sweden; 12 Unit of Research and International Cooperation, University Hospital of Palermo, 90133 Palermo, Italy; 13 Department of Biomedicine, Neurosciences and Advanced Diagnostics (Bind), University of Palermo, 90133 Palermo, Italy; 14 Department of Endocrinology, Singapore General Hospital, Singapore 169856, Singapore; 15 Department of Endocrinology, Diabetes and Metabolic Diseases, University Medical Center Ljubljana, 1000 Ljubljana, Slovenia; 16 Department of Endocrinology, Bharti Hospital & BRIDE, Karnal 132001, India; 17 Department of Endocrinology, Diabetes and Metabolism, Christian Medical College, Vellore 632004, India; 18 Baker Heart and Diabetes Institute, Melbourne, VIC 3004, Australia; 19 Department of Medicine and Oncology, Semmelweis University, 1085 Budapest, Hungary; 20 The Research Institute, Imperial College London Diabetes Centre, Abu Dhabi P.O. Box 48338, United Arab Emirates; 21 Center for Clinical and Epidemiological Research, University Hospital, University of São Paulo, São Paulo 05508-000, Brazil; 22 Diabetes Center, Second Department of Internal Medicine, Democritus University of Thrace, University Hospital of Alexandroupolis, 68100 Alexandroupolis, Greece; 23 Department of Medicine, University of Central Florida College of Medicine, Orlando, FL 32827, USA; 24 Applied Biomedical Research Center, Mashhad University of Medical Sciences, Mashhad 1313199137, Iran; 25 Biotechnology Research Center, Pharmaceutical Technology Institute, Mashhad University of Medical Sciences, Mashhad 1313199137, Iran; 26 Department of Biotechnology, School of Pharmacy, Mashhad University of Medical Sciences, Mashhad 1313199137, Iran; 27 Heart Institute (InCor) University of Sao Paulo Medical School Hospital, São Paulo 05403-900, Brazil; 28 Hospital Israelita Albert Einstein, São Paulo 05652-900, Brazil; 29 Faculty of Medicine, Diabetes, Nutrition and Metabolic Diseases, Carol Davila University, 050474 Bucharest, Romania; 30 Cicarrone Center for the Prevention of Cardiovascular Disease, Johns Hopkins University School of Medicine, Baltimore, MD 21287, USA; 31 Diabetes Research Centre, Chennai 600013, India; 32 Department of Health Promotion, Mother and Child Care, Internal Medicine and Medical Specialties (Promise), University of Palermo, 90133 Palermo, Italy

**Keywords:** periodontitis, periodontal diseases, COVID-19, SARS-CoV-2, comorbidity, risk, non-communicable diseases

## Abstract

Periodontitis is a microbially driven, host-mediated disease that leads to loss of periodontal attachment and resorption of bone. It is associated with the elevation of systemic inflammatory markers and with the presence of systemic comorbidities. Coronavirus disease 2019 (COVID-19) is a contagious disease caused by severe acute respiratory syndrome coronavirus 2 (SARS-CoV-2). Although the majority of patients have mild symptoms, others experience important complications that can lead to death. After the spread of the COVID-19 pandemic, several investigations demonstrating the possible relationship between periodontitis and COVID-19 have been reported. In addition, both periodontal disease and COVID-19 seem to provoke and/or impair several cardiometabolic complications such as cardiovascular disease, type 2 diabetes, metabolic syndrome, dyslipidemia, insulin resistance, obesity, non-alcoholic fatty liver disease, and neurological and neuropsychiatric complications. Therefore, due to the increasing number of investigations focusing on the periodontitis-COVID-19 relationship and considering the severe complications that such an association might cause, this review aims to summarize all existing emerging evidence regarding the link between the periodontitis-COVID-19 axis and consequent cardiometabolic impairments.

## 1. Introduction

Periodontitis is a microbially driven host-mediated disease that leads to loss of periodontal attachment and resorption of bone [1]. During the initial stages, gingival inflammation (i.e., gingivitis) is provoked by bacterial biofilm formation. Consequently, the progression of periodontal disease to destructive periodontitis depends on: microbial dysbiosis in response to nutrients from gingival inflammatory and tissue breakdown products favoring the growth of some bacterial species; and reducing anti-bacterial mechanisms that try to contain the microbial challenge within the gingival sulcus [1]. It has now clearly emerged that periodontal inflammation is not just a local phenomenon, but is also associated with systemic conditions and diseases, such as obesity [2] and diabetes mellitus [3,4,5].

Coronavirus disease 2019 (COVID-19) is a contagious disease caused by severe acute respiratory syndrome coronavirus 2 (SARS-CoV-2). Although in the majority of cases infected patients are asymptomatic and/or have mild symptoms, in some cases this virus can cause severe complications and can be life-threatening. Regrettably, the COVID-19 pandemic heavily hit the regular functionality of the health system worldwide, leading to a tremendous deficiency in providing primary care and leading to an extremely urgent need for new vaccines [6]. A retrospective study conducted on 860 Nepalese COVID-19 patients admitted at the Sukraraj Tropical and Infectious Disease Hospital reported that 5.8% of them died, 2.9% of them needed advanced treatment such as dialysis and mechanical ventilation, and 16% of them showed microbiological evidence of secondary infection [7]. Very interestingly, several serious COVID-19 patients showed the presence of mucormycosis (or black fungus), a severe fungal infection provoked by mucormycetes, probably due to poor oral hygiene, uncontrolled diabetes, unhygienic use of oxygen therapy, and overuse of steroids [8]. Even though the use of steroids has become controversial and confusing among physicians, a recent metanalysis has found that steroids, in particular methylprednisolone, might significantly reduce deaths among hospitalized COVID-19 patients [9]. Despite still being a controversial issue, another study observed that the use of angiotensin-converting enzyme inhibitors/angiotensin II receptor blockers (ACEi/ARB) in COVID-19 hypertensive patients was not associated with their mortality rate [10].

Numerous recent studies highlighted that both periodontitis and COVID-19 might present a bidirectional relationship; in addition, they hypothesized that their synergic effects may lead to an important impairment in patients with other systemic diseases [11,12,13].

Thus, the objective of this review article is to describe the possible association between periodontal disease and COVID-19 with respect to the increased risk and cardiometabolic complications of both diseases.

## 2. Periodontitis and Chronic Non-Communicable Diseases

Chronic non-communicable diseases (NCDs) are diseases that are not directly transmissible from one person to another, as defined by the World Health Organization (WHO). They include cardiovascular diseases, cancer, chronic respiratory diseases, and diabetes, and are responsible for 61% of all deaths worldwide [14].

Periodontitis was reported to be one of the most prevalent chronic NCDs in the USA [15] and has numerous risk factors in common with other NDCs, including smoking, glycemic control, unhealthy diet, alcohol consumption, personal hygiene, stress, genetics, and socio-economic determinants [16].

Periodontal inflammation is not just a local phenomenon. Even though periodontitis has been independently associated with NDCs, the possible relationship between periodontitis and systemic conditions has been widely reported in recent years. In 2013, in the “Proceedings of a workshop jointly held by the European Federation of Periodontology and the American Academy of Periodontology”, the authors concluded that proinflammatory (infectious) events related to periodontal disease may have a systemic impact and, conversely, some systemic disorders might influence periodontal outcomes [17,18,19,20].

A 12-year cohort study in a Korean population reported that periodontally compromised patients have a significantly increased risk of NCDs, in particular obesity (OR 1.30), osteoporosis (OR 1.22), and angina pectoris (OR 1.22) [21]. A cross-sectional study in a Brazilian population of non-smokers and non-alcohol users found that periodontal patients had a higher level of triglycerides, C-reactive protein, blood glucose, lower HDL-c levels, and a higher risk of coronary heart disease [22]. Moreover, older adults had a significantly higher prevalence of dyslipidemia and subclinical atherosclerosis, whereas young adults had a significantly higher prevalence of obesity, pre-diabetes, hypertension, and metabolic syndrome [22]. Although the role of periodontal inflammation as a mediating mechanism in NDCs has not yet been thoroughly explained [23], a very recent overview on oral health and NCDs remarked on the importance of good oral care, which was defined as one of the most important lifestyle-related determinants of health, as well as physical exercise, in reducing the risk of systemic diseases [24]. A systematic review and meta-analysis concluded that periodontitis was associated with an increased risk of all-cause mortality (RR 1.46), mortality due to cardiovascular disease (RR 1.47), cancer (RR 1.38), coronary heart disease (2.58), and cerebrovascular diseases (RR 3.11); pneumonia was associated with edentulism (RR 1.72), which is the ultimate sequela of periodontal disease [25].

Within the aims of the present review, a potential role of periodontitis in the development of respiratory diseases such as asthma, chronic obstructive pulmonary disease (COPD), and pneumonia is investigated [26]. Pneumonia can be classified as community-acquired, developed in non-institutionalized people, nosocomial (hospital-acquired), and/or aspiration pneumonia [27,28]. In particular, nosocomial pneumonia is an infection of the inferior respiratory tract, acquired by patients in a hospital setting at least 48 h after hospitalization [29] due to the aspiration of saliva containing gram-negative anaerobic bacteria (including *Staphylococcus aureus*, *Streptococcus pneumoniae*, *Haemophilus influenzae*, *Pseudomonas aeruginosa*, *Acinetobacter* spp., *Enterobacter* spp.) [30], leading to severe anaerobic pulmonary infections [31]. Therefore, patients with periodontitis may have a significantly increased risk of having pneumonia [32], as confirmed by Awano et al. in a study on a Japanese population of 80-year-olds, which found that aspiration pneumonia caused a mortality 3.9 times higher in subjects with 10 or more teeth with periodontal pockets > 4 mm deep compared with periodontally healthy individuals [33]. Although firm conclusions were not drawn due to different limitations in study design, several systematic reviews have reported that periodontitis might be associated with nosocomial/aspiration pneumonia [34,35], in particular in patients admitted in intensive care units (ICU) and those admitted for a long period (OR 2.55) [36]. Very interestingly, a study on 787 healthy patients (whose data were recollected from the ORIGINS project database) revealed that the log-ratio of *Treponema* to *Corynebacterium* bacteria might be a novel microbial indicator of periodontitis (MIP) that correlates with poor periodontal health and cardiometabolic markers early in disease pathogenesis in both the subgingival plaque and saliva [37].

## 3. COVID-19 and Associated Factors

Coronavirus disease 2019 (COVID-19) is a contagious disease caused by severe acute respiratory syndrome coronavirus 2 (SARS-CoV-2) derived from the Coronaviridae family (WHO).

COVID-19 may infect people mainly via respiratory droplets and aerosols (e.g., from coughing, sneezing, shouting) during close face-to--to-face contact, even though contaminated surfaces might also be contagious [38]. Originally identified in the city of Wuhan in China [39,40,41], COVID-19 provoked a tremendous pandemic with more than 600 million positive cases and more than 6.5 million deaths, as of 9 October 2022 (https://coronavirus.jhu.edu/map.html).

In terms of pathogenicity, SARS-CoV-2 presents a protein spike (S) on its surface that binds to the angiotensin-converting enzyme 2 (ACE2) receptor [42]. Another important role is played by the type 2 transmembrane serine protease (TMPRSS2), which is located in the host cell and leads to viral penetration by cleaving ACE2 and activating the SARS-CoV-2 S protein [42]. ACE2 and TMPRS22 are expressed in host target cells such as alveolar epithelial type II cells [43]. The average incubation period, that is, the time from exposure to symptom onset, is around 5 (2–7) days, and about 97% of subjects who develop any symptoms do so within 11.5 days of infection [44]. However, those features/timings may change depending on the different newer COVID-19 variants that have evolved over time. Patients infected by COVID-19 may be asymptomatic or may either present mild symptoms including anosmia and/or dysgeusia or severe complications that can lead to death [45]. A Chinese study reported an occurrence of mild manifestations in 81% of patients, severe manifestations in 14% of patients, and critical manifestations (e.g., respiratory failure, septic shock, and multiple organ dysfunction [41]) in 5% of the patients [46]. The most common symptoms in hospitalized patients are fever (up to 90% of patients), dry cough (60–86%), shortness of breath (53–80%), fatigue (38%), nausea/vomiting or diarrhea (15–39%), and myalgia (15–44%). Approximately 17%–35% of hospitalized patients with COVID-19 are treated in the ICU, most commonly due to hypoxemic respiratory failure (pneumonia observed in 75% of cases and acute respiratory distress syndrome (ARDS) in 15% of cases) [38,47].

In addition, as described by two review articles, COVID-19 was associated with several oral manifestations, including oral lesions (in both keratinized and non-keratinized mucosa), taste dysfunction (dysgeusia), and xerostomia [48,49]. Dysgeusia was the first recognized oral symptom of COVID-19, whereas aphthous-like lesions, herpetiform lesions, candidiasis, and oral lesions of Kawasaki-like disease were the most common oral manifestations. The most common sites involved were the tongue (38%), labial mucosa (26%), palate (22%), gingiva (8%), buccal mucosa (5%), oropharynx (4%), and tonsil (1%), with those lesions being symptomatic (pain, pruritus, burning sensations) in 68% of cases, equal in both sexes, and more wide-spread and severe in older people with a higher severity of COVID-19 [48]. Lack of oral hygiene, opportunistic infections, stress, immunosuppression, vasculitis, and hyper-inflammatory response were identified as the most important predisposing factors for the onset of oral lesions in COVID-19 patients [48].

## 4. Periodontitis and COVID-19: Association Studies and Outcomes

Over the past two years, several studies with the purpose of demonstrating a connection between periodontitis and COVID-19 have been published.

**Figure 1:**
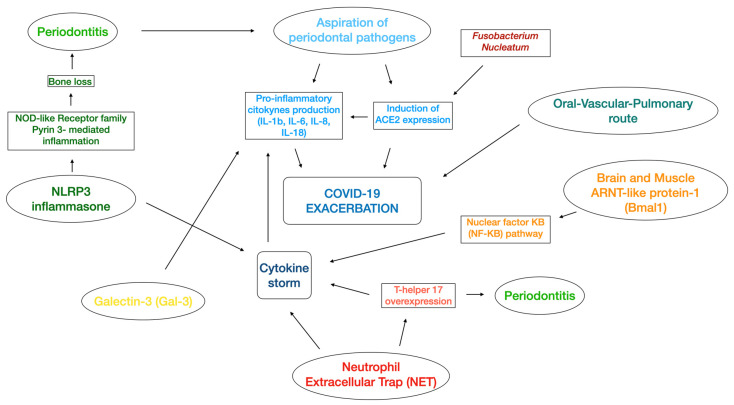
Illustration of the several mechanisms related to the association between periodontitis and COVID-19. NLRP3 inflammasone: nucleotide-binding domain, leucine-rich repeat pyrin domain containing 3 inflammasone; ARNT-like protein 1: aryl hydrocarbon receptor nuclear translocator-like protein 1; IL: Interleukin; NOD-like receptor: nucleotide-binding and oligomineralization domain-like receptor.

In line with the periodontitis–nosocomial pneumonia association discussed above, the aspiration of periodontopathic bacteria induces the expression of angiotensin-converting enzyme 2 (ACE2), a receptor for SARS-CoV-2, and after cleaving the S protein (essential for the development of SARS-CoV-2) by producing proteases [50], may link periodontitis with COVID-19 (Figure 1). In addition, periodontopathic bacteria increase the production of inflammatory cytokines in the lower respiratory tract [50]. An in vitro study confirmed that the culture supernatant of *Fusobacterium nucleatum* (CSF) upregulated the expression of ACE2 in human alveolar epithelial cells and the production of IL-6 and IL-8 in human respiratory epithelial cells [51].

Regarding the role of the mouth, numerous investigations have reported that the oral cavity might be an important viral reservoir [52,53,54,55,56]. Specifically, the virus might be present in the epithelial lining/mucosa wall and periodontal fibroblast of the deep pockets [52]; a high expression of ACE2 was mainly found in the tongue mucosa [55,57] and was higher in the minor salivary glands than in the lungs [58]. Furthermore, other studies have reported that the SARS-CoV-2 viral load was consistently high in saliva (more than in the oropharynx) [59] and was harbored in the dental biofilm [60] and gingival crevicular fluid (GCF) of COVID-19 subjects [61]. Very interestingly, in a postmortem study, video-endoscope minimally invasive biopsies were performed in seven subjects, and the authors found that the periodontal tissues of five patients contained the presence of SARS-CoV-2, indicating that the virus could be stored in the mouth for a long period of time [62].

Another plausible mechanism considers the role of the NLRP3 inflammasome, which are components of the innate immune system and play an important role in the activation of inflammatory responses and maturation and secretion of proinflammatory cytokines, including IL-18 and IL-1β. Several studies have reported that the NOD-like receptor family pyrin 3- (NLRP3-) mediated inflammation was effective in alveolar bone loss [63,64,65]. COVID-19 infection may activate the NLRP3 inflammasome with consequent intensive cytokine expression (cytokine storm) [66] which, in turn, might determine the severity of periodontitis [67,68].

Some authors hypothesized that both diseases might share the production of neutrophil extracellular traps (NET), which resides in an alternative form of cell death (causing damage either directly or via the activation of autoimmune mechanisms), leading to the creation of neutrophil extracellular traps to contain and eliminate insults [69]. The same authors proposed that both diseases may have a connection related to cytokines. In detail, the aforementioned “cytokine storm” (elevated serum level of IL-1b, IL-2, IL-7, IL-8, IL-9 IL-10, IL-17, GM-CSF, G-CSF, INF-gamma, INF alpha, MIP1A, MIP1B, MCP1, and IP1) was present in COVID-19 together with an elevated T-helper 17 (Th17) pathway response [70,71,72]. Interestingly, in advanced periodontal disease, the strong Th17 response might exacerbate the cytokine storm in COVID-19, leading to major complications such as ICU admission, tissue damage in lung infections, and pulmonary oedema [73]. Another possible link might be related to interleukin-6 (IL-6), which was described to be important in the pathogenesis of both periodontitis and the cytokine storm [74,75]. Consistently, a systematic review and meta-analysis reported that, in patients with complicated COVID-19, the mean IL-6 levels were 2.9-fold higher compared with patients presenting non-complicated disease [76].

Other authors proposed the “Oral-Vascular-Pulmonary Route”, which consists of a direct viral delivery from the oral cavity (gingival capillaries) via the venous drainage of the mouth, neck (jugular veins), and chest (superior vena cava) through the right side of the heart and then to the pulmonary vessels, causing pulmonary vasoconstriction and immunothrombosis [77]. Poor oral hygiene and periodontal disease (with the consequent presence of periodontal wounds due to ulcerations in the pocket’s epithelium) may thus increase the probability of having severe COVID-19 complications [77].

Another studied mechanism involves the role of galectin-3 (Gal-3), a member of the beta-galactoside binding protein family with proinflammatory properties and involved in T-cell-mediated inflammation that is expressed in immune cells, epithelial cells, endothelial cells, and sensory neurons [78]. A study demonstrated that a consistent area in the spike protein (essential for virus entrance into the host cells) of COVID-19 is almost the same as the morphology of Gal-3 [79], whereas other authors reported that there is a positive correlation between increased levels of Gal-3 and the severity of periodontitis (data not published) [78]. Therefore, the authors hypothesized that Gal-3-mediated increased immune response and increased viral attachment might reinforce the relationship between periodontitis and COVID-19 [78].

A further link may derive from the circadian system [80]. In fact, a circadian gene named brain and muscle ARNT-like protein-1 (Bmal1), which decreases when the sleep-wake cycle is disrupted, may induce the cytokine storm (common for both COVID-19 and periodontitis) via the nuclear factor kB (NF-kB) pathway [80].

Figure 1 illustrates the described perio-COVID-19 mechanisms, and Table 1 summarizes all of the possible molecules that are involved.

A case-control study on 568 Qatarian patients with COVID-19 reported that, after adjusting for important confounders including smoking, age, and sex, periodontitis was associated with important COVID-19 complications (overall OR 3.67, 95% CI 1.46–9.27) such as intensive care unit (ICU) admissions (OR 3.54, 95% CI 1.39–9.05), need for assisted ventilation (OR 4.57, 95% CI 1.19–17.4), and death (OR 8.81, 95% CI 1.00–77.7). Moreover, C-Reactive Protein, white blood cells (WBC), and HbA1c levels were significantly higher in COVID-19 patients with periodontitis [81]. Another case-control study in India observed that, after adjusting for frequency of self-oral hygiene and age, COVID-19 was significantly associated with mean plaque score ≥ 1 (OR 7.01, 95% CI 1.83–26.94), gingivitis (OR 17.65, 95% CI 5.95–52.37), mean clinical attachment level (CAL) ≥ 2 mm (OR 8.46, 95% CI 3.47–20.63) and severe periodontitis (OR 11.75, 95% CI 3.89–35.49); additionally, gingival bleeding and plaque accumulation were more frequently present in COVID-19 patients [82]. A further case-control study in an Indian cohort showed that patients with bleeding on probing presented a greater risk of a need for assisted ventilation (OR 4.14, 95% CI 1.51–11.34, in particular among patients with periodontitis Stage III-IV), presence of COVID-19 pneumonia (OR 3.63), and hospital admission (OR 3.18, 95% CI 1.24–8.15) [83]. A higher severity of periodontitis was correlated with the need of assisted ventilation (OR 7.45), COVID-19 pneumonia (OR 4.42), death (OR 14.58), and hospital admission (OR 36.52). Furthermore, 10% of deceased patients had severe periodontal disease [83]. However, it must be highlighted that significant confounders such as socioeconomic status, obesity, and smoking were not considered.

A retrospective study based on examination records and panoramic X-rays showed that patients with a higher dental damage stage (DD Stg) (a new score that takes into account periodontal condition, caries, root canal treatment, and missing teeth) presented an increased hospitalization and mortality rate of COVID-19 [84]. Nevertheless, no confounding variables from other factors were taken into account in the analysis. Two more retrospective studies reported that patients with COVID-19 and periodontitis presented a significantly higher mortality (OR 1.71, 95% CI: 1.05–2.72) compared with periodontally health patients after adjusting for age, sex, ethnicity, average total household income, BMI, systolic and diastolic blood pressure, history of smoking, and history of previous systemic conditions [85]; additionally, patients with periodontal diseases were 4.7 times more likely to develop COVID-19 compared with non-periodontal patients after adjusting for smoking [86]. A Mendelian randomization (MR) study suggested that genetically proxied periodontal disease was significantly associated with a major risk of susceptibility and hospitalization of COVID-19, even though it did not seem to have a causal effect on COVID-19 severe respiratory complications [87].

Different reviews have indicated a series of possible shared risk factors such as aging, male sex, diabetes [3,4,50], hypertension and cardiovascular disease, coronary artery disease, atherosclerotic diseases, obesity [2], pregnancy, chronic obstructive pulmonary disease (COPD), smoking, asthma, HIV, cancer, oral dysbiosis, liver diseases, dementia, chronic kidney disease, Down syndrome, type A blood group, specific ethnic groups, physical disability/learning difficulty, and rheumatoid arthritis [77,88,89]. A very recent meta-analysis of epidemiological studies found that periodontal patients with COVID-19 have a four times higher risk of hospitalization, a six times higher risk of need for assisting ventilation, and seven times higher risk of death due to COVID-19 complications [90].

Oral hygiene has been hypothesized to be crucial in preventing post-COVID-19 complications [91]. Metagenomic analysis of COVID-19 patients showed the presence of high reads for periodontal bacteria such as *Fusobacterium, Prevotella,* and *Staphylococcus* [92]. Authors recommend that self-oral hygiene should be improved to reduce the bacterial load and the risk of bacterial superinfection [91]. A cross-sectional study on an Egyptian population, with the aid of questionnaires, found that poor oral status significantly impacted the severity of COVID-19 and, additionally, was correlated with increased values of C-reactive protein (CRP) and delayed recovery period [93]. Curiously, an online survey in a Turkish population showed an association between COVID-19 fear and oral health status [94]. Patients that started to pay more attention to their hygiene habits and rate/quality of food consumption were the ones with the higher fear of COVID-19; moreover, although those patients had increased complaints and dental problems, they hesitated to go to the dentist as they believed that the dental setting might increase the risks of COVID-19 contamination [94].

Conversely, other authors focused on the plausible negative effect that COVID-19 might have on periodontal health and hypothesized that COVID-19 might predispose to necrotizing periodontal disease (NPD) [95]. In fact, bacteria that are usually present in NPD lesions such as *Prevotella intermedia*, *Fusobacterium,* and *Treponema* were found in metagenomic analyses of infected subjects with severe COVID-19 disease [95].

Table 2 summarizes the existing studies focused on the plausible periodontitis–COVID-19 relationship.

## 5. Cardiometabolic Connections in the Perio-COVID-19 Axis

As extensively reported in the literature, periodontitis and cardiometabolic diseases (including cardiovascular disease, type 2 diabetes, metabolic syndrome, dyslipidemia, insulin resistance, obesity, and non-alcoholic fatty liver disease [96]) might present a bidirectional relationship [97,98,99]. The dysbiosis of periodontal microbiota might cause endotoxemia as well, as it occurs due to the entrance of bacterial lipopolysaccharide (LPS) into the blood circulation [100]. Consequently, those bacteria in the blood stream may provoke inflammation [101], formation and/or rupture of atherosclerotic lesions [102], and may affect the vessel walls (i.e., endothelial dysfunction, [103]), leading to a major risk of cardiometabolic complications [100]. A retrospective cohort study based on a UK population found that subjects with gingivitis and/or periodontitis had an increased likelihood of having a diagnosis of cardiometabolic diseases (aOR: 1.16; 95% CI 1.13 to 1.19) and an increased risk of developing cardiometabolic diseases (HR 1.07; 95% CI 1.03 to 1.10) compared with periodontally healthy patients [104].

**Figure 2:**
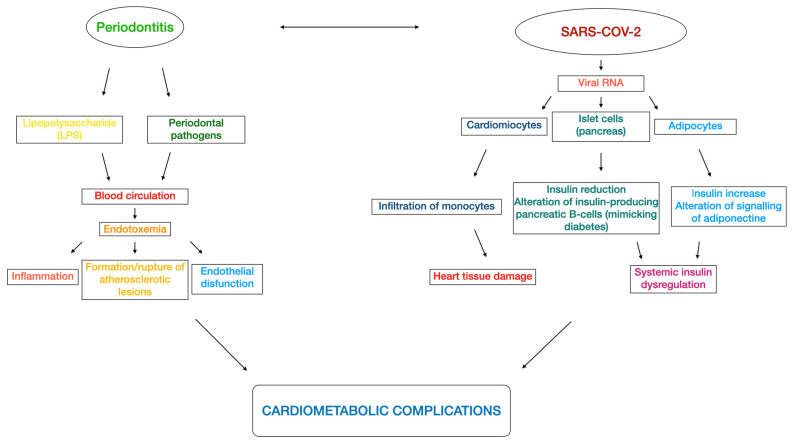
Flowchart of the several perio-COVID pathways that might lead to cardiometabolic impairments.

Similarly, as suggested by the American College of Cardiology Foundation, COVID-19 showed epidemiological and mechanistic relationships with cardiometabolic links [105,106] such as abnormal adiposity, dysglycemia [107,108,109,110,111], dyslipidemia, and hypertension [112]. In fact, COVID-19 caused an inflammatory response that might affect the cardiometabolic status. More specifically, as reported by a study using a hamster model, SARS-CoV-2 infected cardiomyocytes and provoked a mechanism of immune cell infiltration and histopathology that might result in heart tissue damage in COVID-19 patients [113]. Moreover, evidence suggests that altered insulin signaling with consequent insulin dysregulation was caused by the presence of viral RNA in pancreatic cells and adipocytes [114,115]. A Swedish nationwide case-control study of COVID-19 patients requiring invasive mechanical ventilation reported that diabetes, hypertension, and obesity were independently associated with severe COVID-19; additionally, the younger population (<57 years) had a stronger association than the older one [116]. Interestingly, an investigation based on data from the UK Biobank found that increased adiposity (adipose volume), assessed as body mass index (BMI), waist:hip ratio (WHR), and body fat, was associated with higher risks of COVID-19-related mortality [117]. In addition, some COVID-19 patients unfortunately presented the so called “long-COVID” (also named “long-haul COVID”, “post-COVID syndrome”, or “post-COVID conditions”), characterized by protracted symptoms for a period that may remain from more than 4 weeks to more than 3 months [118,119]. In detail, those patients showed malaise, fatigue, dyspnea, neuropsychiatric syndromes, and defect in memory and concentration [120]. A UK cohort study with 12-month follow-up reported that diabetes incidence was elevated for at least 12 weeks following COVID-19 before decreasing, whereas cardiovascular disease incidence such as pulmonary embolism, atrial arrhythmias, and venous thromboses was high for the first 4 weeks, then decreased from 5 to 12 weeks, and, lastly, consistently dropped from 13 to 52 weeks [121]. Figure 2 illustrates the plausible perio-COVID pathways that might impair the cardiometabolic complications.

## 6. Clinical Relevance and Take-Home Message

Due to the aforementioned concrete risks of developing severe complications, a syndemic approach involving biological, social, economic, and environmental factors has been proposed by the Cardiometabolic Panel of International experts on Syndemic COVID-19 (CAPISCO) [122]. Furthermore, physicians should follow a holistic approach when treating COVID-19 patients, also taking into account the observed increased neurological and neuropsychiatric complications such as memory loss, sleep disorders, impaired concentration, major depression, and delirium [123,124].

Moreover, efforts should be taken to prevent periodontal disease and, for patients that already have periodontitis, an immediate intervention should be provided to improve their gingival health. In particular, physicians should remark the importance of having a proper oral hygiene, and they should ideally suggest to their patients to have regular dental visits in order to prevent or detect any form of periodontal disease, above all during the current pandemic period, keeping in mind the potential associations between periodontitis and cardiometabolic complications [125,126,127].

## 7. Conclusions and Recommendations for Future Studies

Within the limitations of the included studies, the following conclusions can be drawn:

- Periodontitis and COVID-19 have in common a hyper-inflammatory state. Despite some consistent associations in cross-sectional studies, definitive conclusions regarding their link cannot be drawn.
- Nevertheless, in light of a possible bidirectional relationship, the importance of maintaining or achieving periodontal health to prevent or contain COVID-19 complications must be highlighted.
- The importance of having proper oral hygiene to reduce any possible viral source proceeding from the mouth must be considered and, in particular, periodontally compromised patients should receive adequate periodontal management, including oral hygiene instructions.
- A multi-professional therapeutic plan from physicians and dentists must be agreed upon to identify and adequately manage each COVID-19 patient while doing so in a tailored manner.

In terms of future research, long-term clinical trials investigating the magnitude of the association between periodontitis and COVID-19 and their mechanisms are needed.

## Figures and Tables

**Table 1:** Summary of some of the several molecules that are involved in the mechanisms behind the COVID-19 and the periodontitis relationship.

Molecules Involved in the Perio-COVID-19 Axis
Angiotensin-converting enzyme 2 (ACE2), S protein [50]

Interleukin-6 (IL-6), interleukin-8 (IL-8) [51,76]

NLRP3 inflammasome, Interleukin-18 (IL-18); interleukin-1β (IL-1β) [65,66]

Neutrophil extracellular traps (NET) [69]

T-helper 17 (Th17) [71,72]

Galectin-3 (Gal-3) [78,79]

ARNT-like protein-1 (Bmal1), nuclear factor kB (NF-kB) [80]

ARNT-like protein-1: aryl hydrocarbon receptor nuclear translocator-like protein 1; NLRP3 inflammasone: nucleotide-binding domain, leucine-rich repeat pyrin domain containing 3 inflammasone.

**Table 2:** Summary of existing studies investigating the possible association between COVID-19 and periodontitis.

Authors	Study	Periodontitis Definition	COVID-19	Confounders	Findings
Marouf et al., 2021 [81], 568 patients	Case-control	Periodontally healthy or initial periodontitis (Stages 0–1): bone loss less than the coronal third of the root length (15%) in OPGs, or ≤2 mm in bitewing radiographs Periodontitis (Stages 2–4): bone loss more than the coronal third of the root length (>15%) in OPGs, or >2 mm in bitewing radiographs	Two subsequent positive polymerase chain reaction (PCR) tests	Analysis adjusted for smoking, age and sex	Periodontitis was associated with intensive care unit (ICU) admission, need for assisted ventilation, and death C-Reactive Protein, white blood cells (WBC), and HbA1c levels were significantly higher in presence of periodontitis.
Anand et al., 2021 [82], 150 patients	Case-control	Gingivitis and Periodontitis (2017 World Workshop)	Positive real-time (rRT-PCR) test	Analysis adjusted for frequency of self-oral hygiene and age	COVID-19 was significantly associated with mean plaque score ≥ 1, gingivitis, mean clinical attachment level (CAL) ≥ 2 mm, and severe periodontitis. Moreover, gingival bleeding and plaque accumulation were more frequently present in COVID-19 patients.
Gupta et al., 2021 [83], 82 patients	Case-control	Gingivitis and Periodontitis (2017 World Workshop)	Nasopharyngeal swab (NPS) test	No potential confounders were considered	Patients with bleeding on probing presented more risks of need for assisted ventilation, suffering from COVID-19 pneumonia, and hospital admission. Higher severity of periodontitis was correlated with need for assisted ventilation, COVID-19 pneumonia, death, and hospital admission 10% of deceased patients had a severe periodontal disease.
Sirin and Ozcelik 2021 [84], 137 patients	Retrospective	Radiographic bone loss (RBL) classification	History of COVID-19: positive real-time PCR test	No potential confounders were considered	Patients with a higher dental damage stage (DD Stg) presented an increased hospitalization and mortality rate of COVID-19.
Larvin et al., 2020 [85], 13,253 patients	Retrospective	Mild-to-moderate periodontitis: self-reported oral health indicators of bleeding and painful gums; severe periodontitis: loose teeth	Positive test results (not specified) taken by the UK Biobank records	Analysis adjusted for age, sex, ethnicity, average total household income, BMI, systolic and diastolic blood pressure, history of smoking, and history of previous systemic conditions	Patients with COVID-19 and periodontitis presented a significantly higher mortality compared with periodontally health patients.
Katz et al., 2020 [86], 889 patients	Retrospective	Not reported	Positive test results (not specified) taken by the University of Florida patient registry	Analysis adjusted for smoking	Patients with periodontal diseases were 4.7 times more likely to develop COVID-19 compared with non-periodontal patients.
Wang et al., 2021 [87], 1,299,010 patients	Mendelian randomization	Not reported	-	Not specified	Genetically proxied periodontal disease was significantly associated with a major risk of susceptibility and hospitalization of COVID-19. However, no causal effect on COVID-19 severe respiratory complication was found.
Kamel et al., 2021 [93], 464 patients	Cross-sectional	Not reported	Positive PCR tests	No potential confounders were considered	Poor oral status significantly impacted the severity of COVID-19 and was additionally correlated with increased values of C-reactive proteins (CRP) and delayed recovery period.
